# Media frame development of direct air capture 2011–2023: A comparative analysis of Europe and North America

**DOI:** 10.1016/j.isci.2024.111360

**Published:** 2024-11-12

**Authors:** Paul J. Upham, Emina Ibrahimović

**Affiliations:** 1 Integrated Research on Energy, Environment and Society, ESRIG, Nijenborgh 6, 9747 AG Groningen, the Netherlands

**Keywords:** Engineering, Materials science engineering, Social sciences

## Abstract

Media portrayal of direct air capture (DAC) matters for public opinion and policy. How is DAC framed in the North American and European press, are there differences between the two, and what are the implications for public perceptions of DAC? This study provides an insight into the media frames of DAC in North American and European news articles, for the period 2011–2023. With no prior work on this topic, five generic media frames were used for classification purposes. While the study is intended to be exploratory rather than definitive, European articles were found to be more critical than North American articles, more often viewing DAC as an expensive mitigation option. North American articles highlighted the lack of government financial support for DAC. The employment and economic potential of DAC was acknowledged in both newspaper datasets. Although DAC is relatively little framed in relation to human interest, morality, or conflict, these frames may be important in shaping public opinion in future.

## Highlights

• Generic media frames of DAC in North American and European news articles 2011–2023
• European articles more critical than North American articles, portraying DAC as expensive
• North American articles highlighted lack of government financial support for DAC
• Little use of human interest, morality, or conflict frames; of potential, future importance

## Graphical abstract

**Figure:**
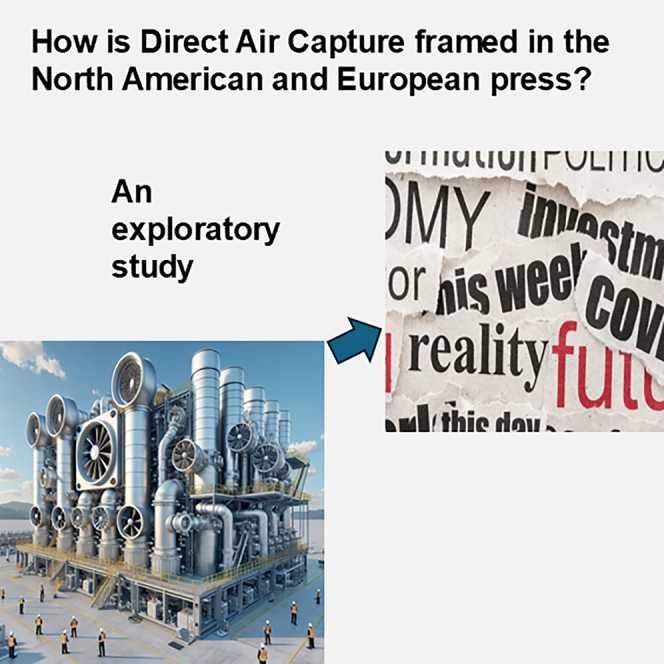

## Introduction

In 2018, Intergovernmental Panel on Climate Change (IPCC) recommended removal of 10 GtCO_2_ annually,^1^ for which purpose direct air capture (DAC) uses engineered processes to chemically or physically extract carbon dioxide directly from the atmosphere.^2^ The concentrated carbon dioxide can be either stored or re-used for products and other applications.^3^ While DAC is generally acknowledged to have a high carbon abatement cost,^4^ several countries have instituted supportive policy frameworks including subsidies or tax credits.^5^

Here, we examine portrayal of DAC specifically in digitized print and online news media, with the rationale that media framing both shapes and reflects public perceptions, and has the potential to influence both policy and investment. In short, media framing of technologies, especially new technologies, plays a role in how, and how quickly, those technologies develop. For this purpose, we draw on Erwin Goffman’s Media Frame Theory,^6^ which argues that the mass media function as a platform for presenting information to audiences within specific frames or perspectives.

While the nature of the mass media has changed considerably over the last decades, framing in the sense of drawing attention to particular aspects of a phenomenon is generic to communication. Hence a frame is a “conceptual tool which media and individuals rely on to convey, interpret, and evaluate information.”^7^ Drawing on Goffman’s theory, this study aims to identify and trace the evolution of media frames of DAC in Europe and North America over the time period of 2011–2023, the start date being the first instance of a DAC article in the news database used.

Although there are public perceptions studies of carbon dioxide removal,^4^ which we consider further in the discussion section in addition to other relevant studies, we are not aware of other DAC news framing studies. Given this, we apply media frames known to capture most types of news article or report^8^: attribution of responsibility, human interest, conflict, morality, and economic consequences (explained further in the procedures section). Such *a priori* categorization or coding has the value of mapping the frame terrain in terms that are familiar to frame analysts across topics, supporting generalized observations.

Frame analysis is used to characterize how aspects of phenomena are highlighted, hence providing an insight into the ways in which phenomena are perceived in the public sphere. These perceptions matter because they can influence policy and investment decisions, including for new technologies. DAC is one such set of technologies. DAC removes carbon from the atmosphere, but the extent of its value is sometimes contested. We wanted to know whether this contestation is widespread in newspapers, which we take as an example of news media. We compare framing of DAC in North American and European newspapers. As the number of newspaper articles on this topic is large, and as there is little published work on DAC framing, we took a 10% sample and used media frames known to capture most news frames. As a caveat, it should be noted that frame analysis is not an exact science, but involves a degree of coder subjectivity and the real possibility of changes to newspaper databases over time. Given this, our findings should be viewed as indicative, exploratory and for the purpose of discussion, not as fixed over time and definitive. Nonetheless, in the sample examined, we found greater skepticism of DAC in the European press, but we also found that DAC was not (yet) subject to a high prevalence of conflict or morality frames. We think that these latter frames may matter for public perceptions in future.

## Results

### News frame incidence over time

**Figure 1:**
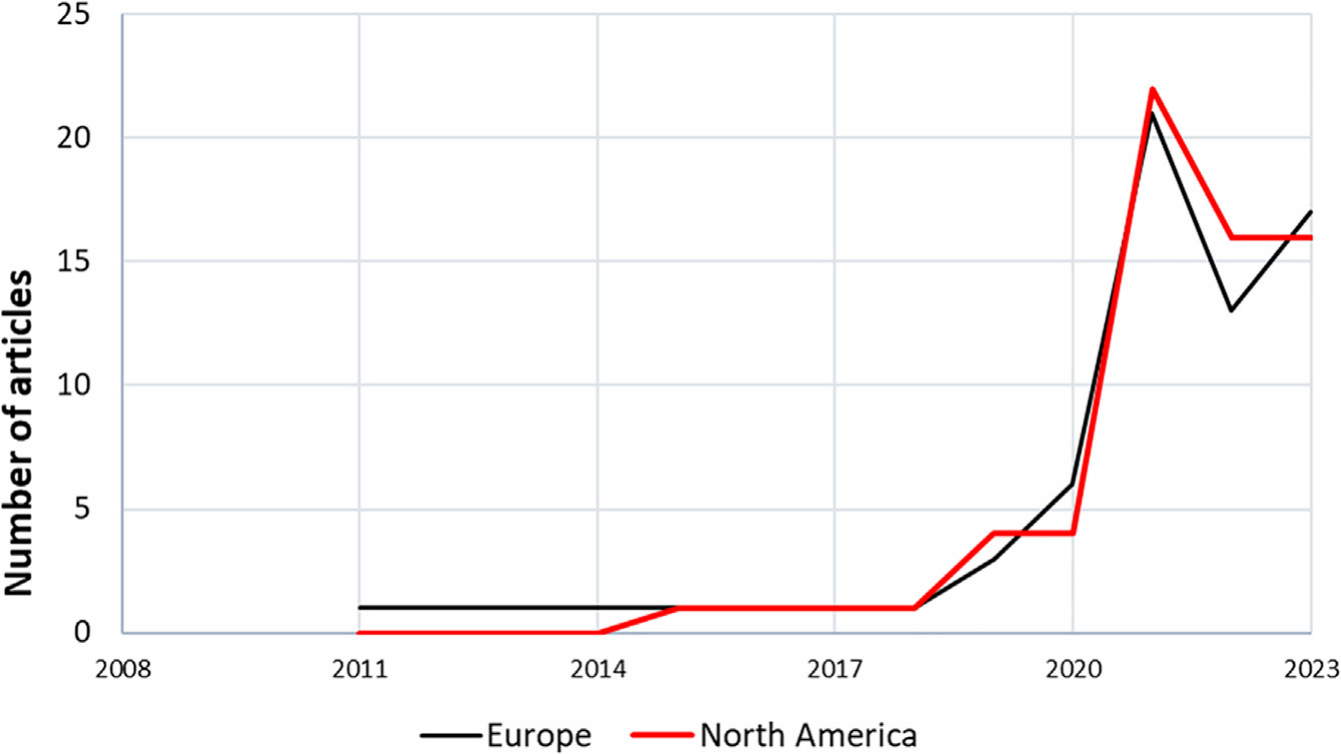
Article publication per year 2011–2023 for Europe and North America

**Figure 2:**
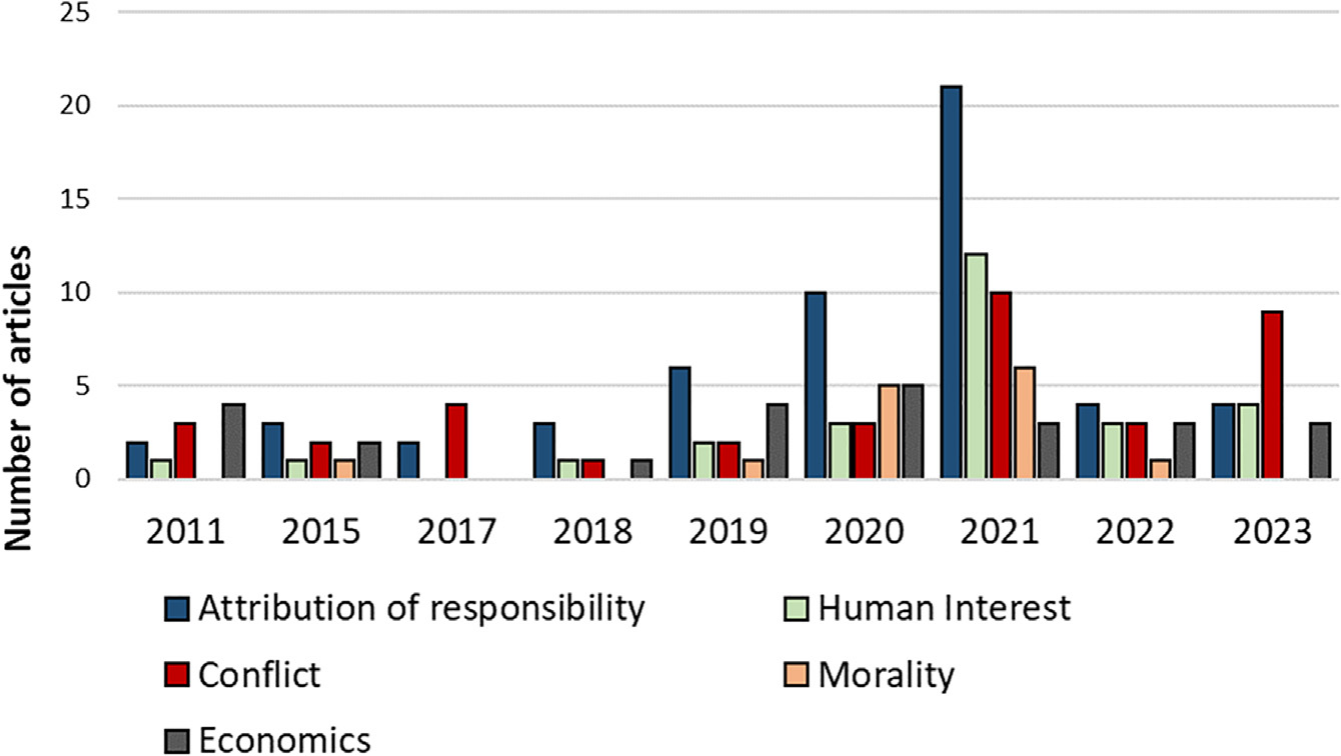
Distribution of news media frames over the time period of 2011–2023 in Europe Note: Frequency is the cumulative frame mentions per year per news media frame.

**Figure 3:**
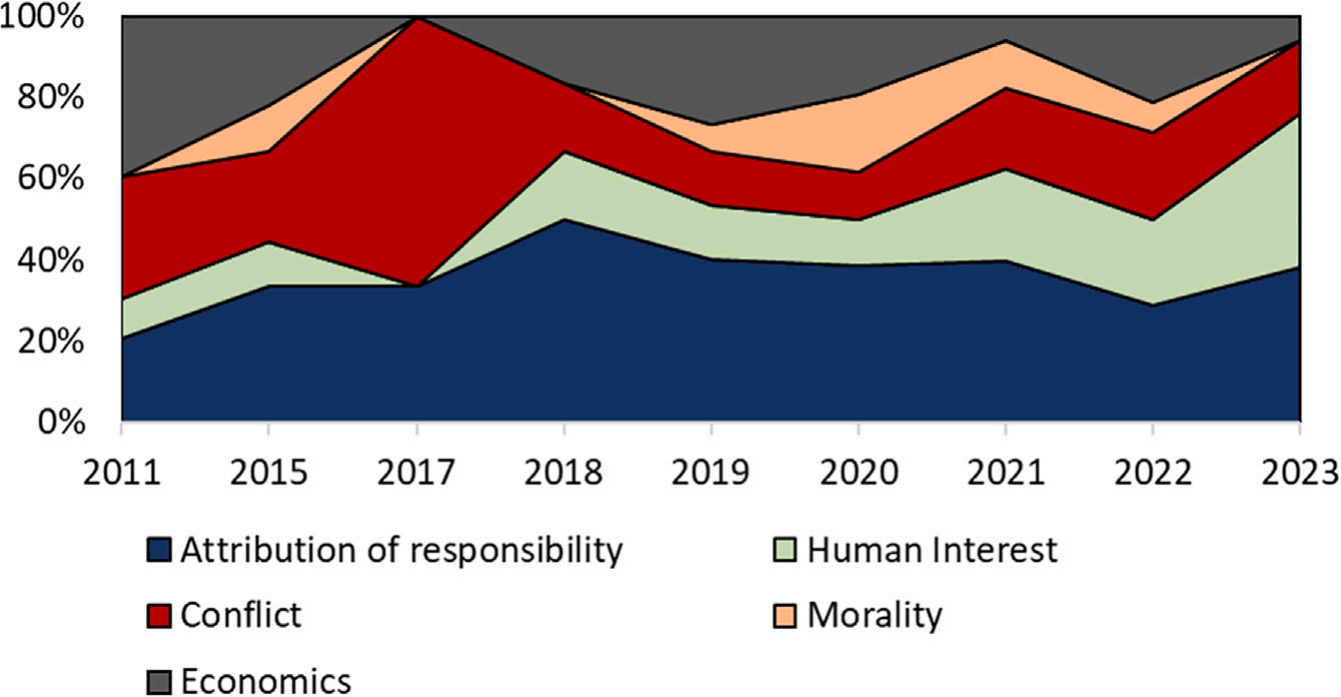
Percentage distribution of news media frames over the time period of 2011–2023 in Europe Note: Percentages relate to the number of published articles each year.

**Figure 4:**
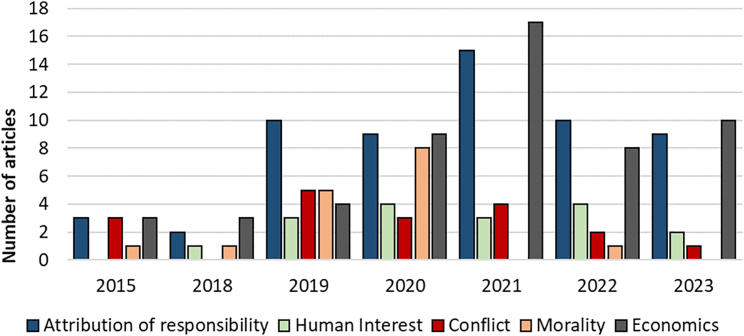
Distribution of news media frames over the time period of 2015–2023 in North America Note: Percentages relate to the number of published articles each year.

**Figure 5:**
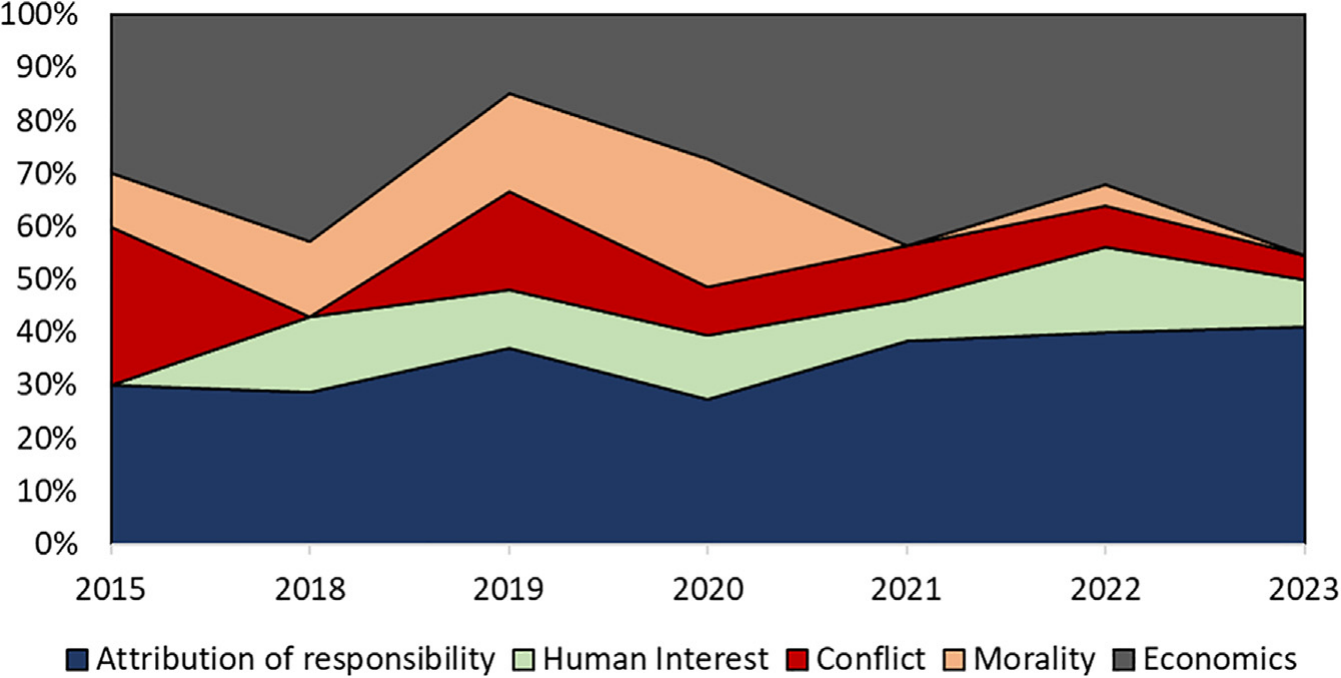
Normalized distribution of news media frames over the time period of 2015–2023 in North America Note: annual percentages are referenced to annual frequencies, to account for the differing number of articles published each year.

Keeping in mind our caveat about the indicative nature of quantification in this context, the appendices and supplemental data provide further qualitative and quantitative detail for all of the results. In the main article text, Figures 1, 2, 3, 4, and 5 show the news frame incidence over time for Europe and North America separately. Figure 1 shows newspaper article publication frequency referencing DAC for the years 2011–2023. In Europe, the first news article on DAC was published in 2011, while the first North American article came four years later in 2015. Figure 2 shows the distribution of news media frames per year across the years 2011–2023 for Europe. Frequency is the total number of occurrences of a frame in the total number of articles in that specific year. Most notable for Europe are an increase in the frequency of the attribution of responsibility frame (i.e., DAC in the context of concern with climate change generally) until a peak in 2021, and a lower but general peak of most frames in 2021, likely reflecting anticipated IPCC consideration of Carbon dioxide removal (CDR) and DAC in its Sixth Assessment Report.^9^ Figure 3 accounts for the changing number of articles annually and shows the relative incidence of the different frames over time in Europe, with the attribution of responsibility frame being the most prevalent.

Figure 4 shows the distribution of news media frames per year for North America (2015–2023), with DAC being referenced most frequently in the context of attribution of responsibility and economic consequence frames. Figure 5 accounts for the changing number of articles annually and shows the relative incidence of the different frames over time in North America, with the economic consequences frame being predominant throughout. Besides economic consequences, the attribution of responsibility frame consistently stands out as relatively prominent.

### Frame indicators: Europe and North America compared

#### Attribution of responsibility

**Figure 6:**
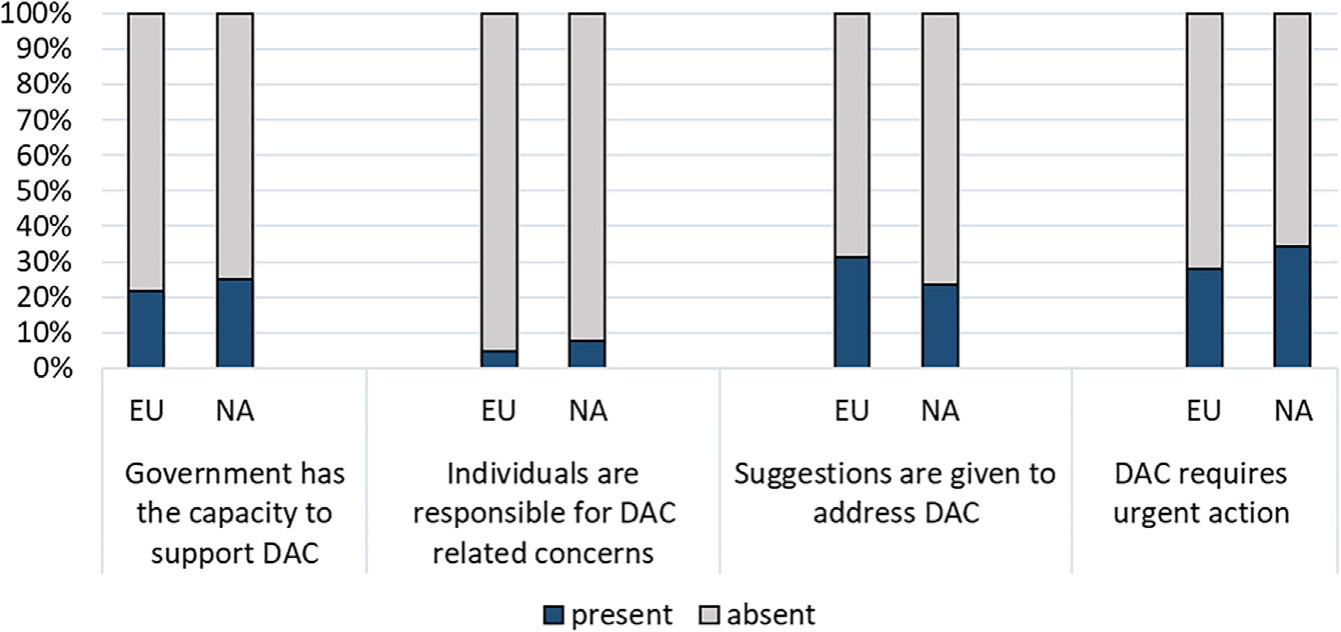
Attribution of responsibility: Percentage of frame indicators for Europe and North America Note: for Tables 6, 7, 8, 9, and 10, the percentages relate to the total set of frame indicators, on a per continent basis.

For Europe and North America, Figure 6 and appended Tables A1a and A1b show the relatively high prevalence of attribution of responsibility frame indicators, graphed as percentages of the total frame indicator set (qualitatively listed in Table 1). For both continents, a larger percentage of the articles attribute responsibility to governments rather than individuals. European news articles focus primarily on the lack of governmental action to reduce climate change and to implement DAC. North American articles focus more on the lack of governmental financial support for DAC. A large proportion of articles in this frame urge governmental support and action for DAC.

**Table 1:** Frames and frame indicators based on direct air capture-related concerns

1. Attribution of responsibility
a. Does the story suggest that some level of the government has the capacity to address DAC-related concerns?
b. Does the story suggest that particular individuals or groups are responsible for DAC-related concerns?
c. Does the story suggest strategies or actions to address DAC-related concerns?
d. Does the story suggest that DAC-related concerns require urgent action?
2. Human interest
a. Does the story provide a human example or “human face” for DAC-related concerns?
b. Does the story refer to the private or personal lives of actors involved?
c. Does the story emphasize how individuals and groups are affected by DAC-related concerns?
d. Does the story acknowledge the diversity of opinions and perspectives within the DAC-related concerns?
3. Conflict
a. Does the story reflect disagreement between parties- individuals-groups-countries?
b. Does one party-individual-group-country reproach another?
c. Does the story refer to two sides or to more than two sides of the DAC-related concern?
d. Does the story refer to winners and losers?
4. Morality
a. Does the story contain any moral messages?
b. Does the story make reference to morality, God, and other religious tenets?
c. Does the story offer specific societal values or ethical guidelines when considering DAC-related decisions?
d. Does the story discuss consequences of certain actions or choices in terms of *right* and *wrong*?
5. Economic consequences
a. Is there reference to financial losses or gains now or in the future?
b. Is there reference to costs or the degree of expense involved?
c. Is there a reference to the economic consequences of pursuing or not pursuing a course of action?
d. Does the story consider economic viability/feasibility regarding the implementation of DAC?

The frames are adapted from Semetko and Valkenburg (2000).^8^

#### Human interest

**Figure 7:**
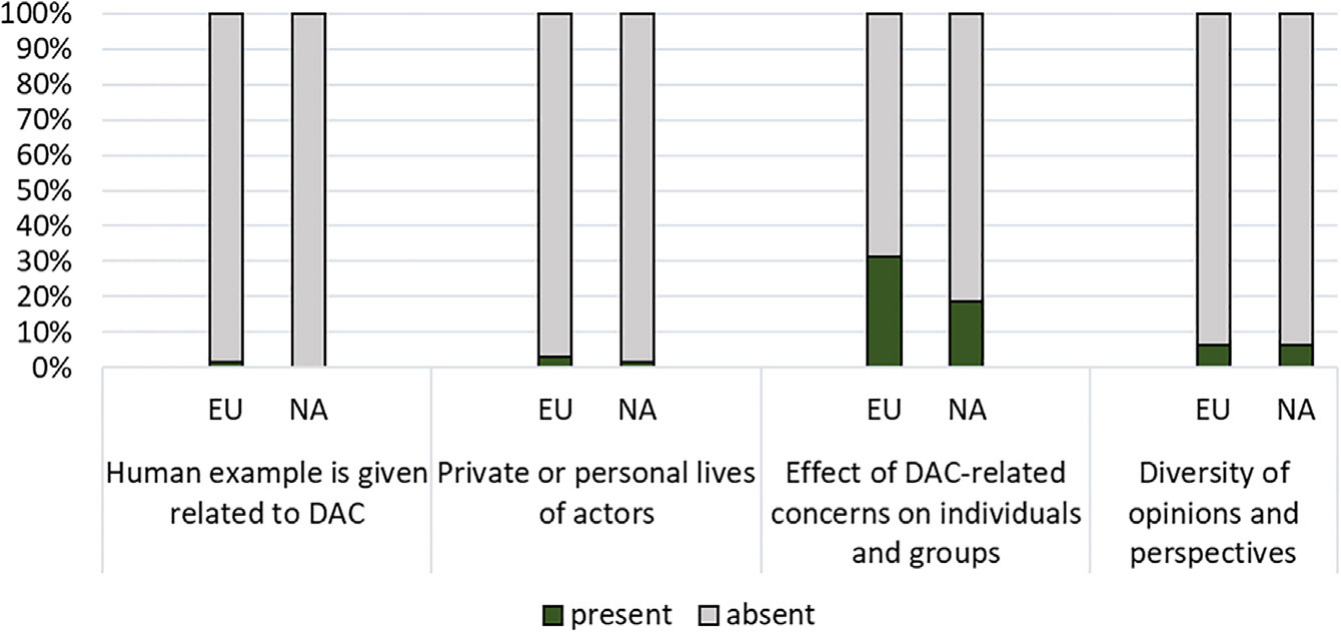
Human interest: Percentage of frame indicators for Europe and North America

Figure 7 and appended Tables A2a and A2b show the prevalence of DAC-related human interest in the media coverage of Europe and North America, which mostly takes the form of the potential effects of DAC on individuals and groups, via its climate mitigation and economic potential. There are more such frames in European articles, composed primarily of references to job creation and job protection. The North American coverage is similar but broader, referring to the relationship between DAC and jobs, health and the economy as a whole.

#### Conflict

**Figure 8:**
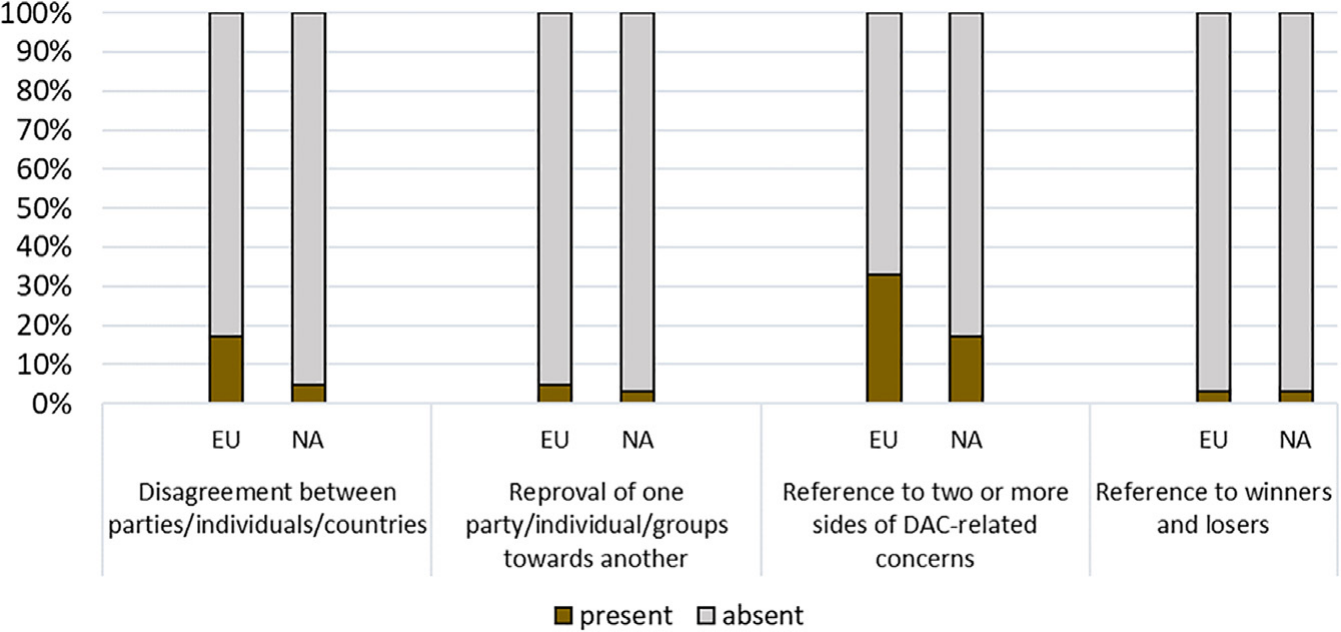
Conflict: Percentage of frame indicators for Europe and North America

Conflict here refers to disagreement about DAC per se and in terms of its carbon abatement value, as well as its distributional consequences. This frame is found relatively infrequently, but Figure 8 shows its higher prevalence in European articles. Essentially, more European articles than North American are critical or skeptical of DAC, particularly for its high carbon abatement cost. Appended Tables A3a and A3b provide illustrative quotations.

#### Morality

**Figure 9:**
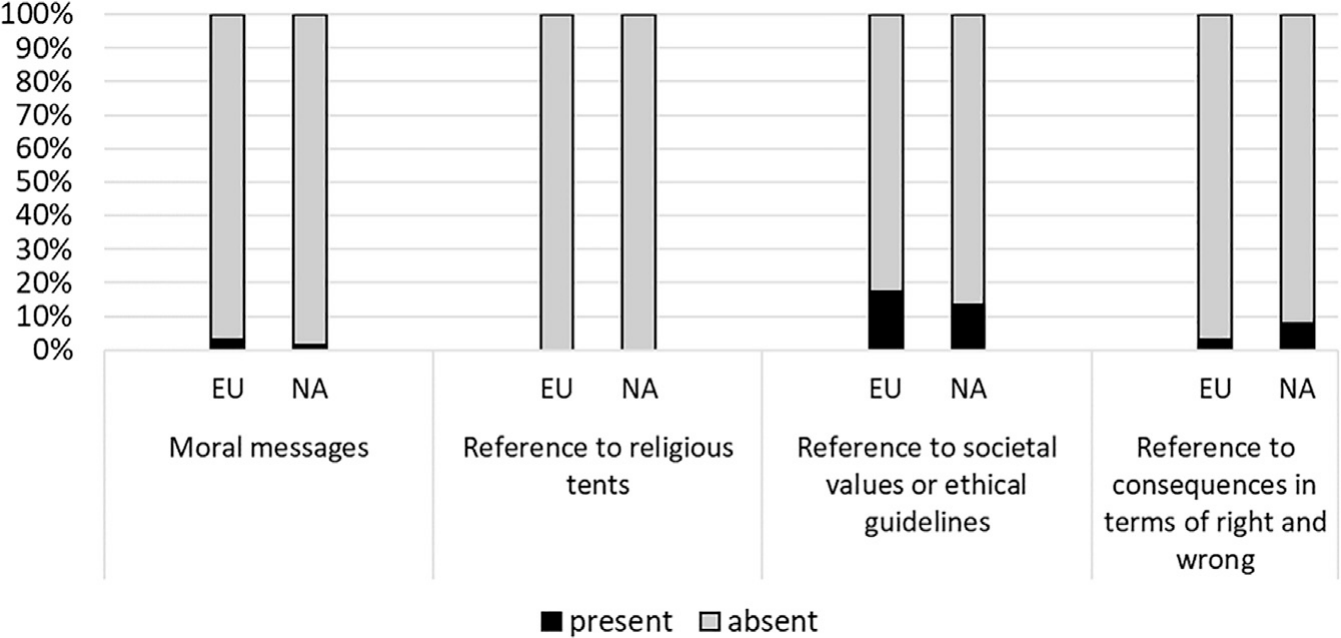
Morality: Percentage of frame indicators for Europe and North America

Figure 9 shows the low prevalence of the morality frame in the newspaper coverage of DAC: moral messages and references to religious tenets are negligible. A small fraction of European articles refer to the principle that industrial societies should go beyond the reduction of carbon emissions and toward their active removal (also appended Table A4a). A small fraction of North American articles refer to the principle that those responsible for carbon emissions should accept the responsibility of carbon mitigation (also appended Table A4b). North American articles also advocate international collaboration to support immediate investment in DAC, and frame DAC as a form of public utility.

#### Economic consequences

**Figure 10:**
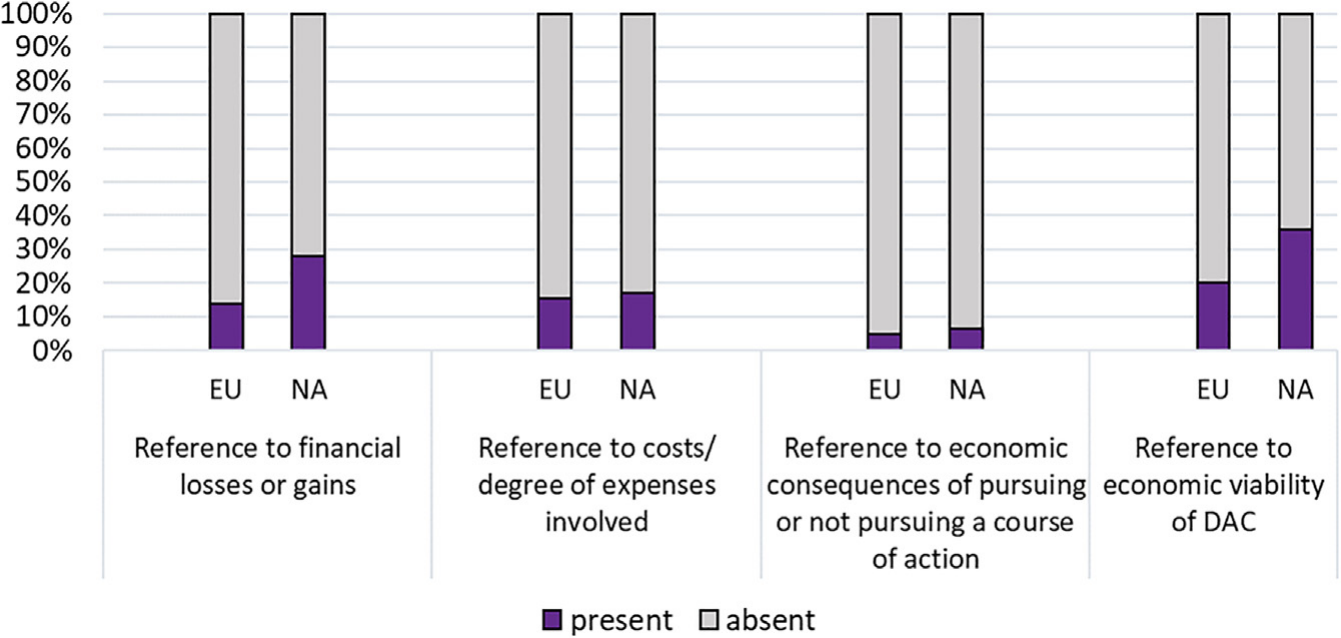
Economic consequences: Percentage of frame indicators for Europe and North America

Figure 10 relates to the prevalence of frames that concern the economic consequences of DAC, including loss, gains, cost and economic viability. All are more prevalent in North American than European articles. Where the frame is present in European articles, this mostly relates to the high abatement cost of DAC and the expectation that this will continue (also appended Table A5a). North American articles are more optimistic, on condition of adequate funding and fiscal support (appended Table A5b). Articles on both continents also refer to the wide variation in DAC carbon abatement costs.

## Discussion

Media framing is particularly important in environmental contexts, where it plays a major role in shaping how the public understands and perceives environmental challenges such as climate change.^10^ The notably increased incidence in news framing of DAC is relatively recent,^11^ as also found in work on tweet incidence for attention to CDR.^12^ Both reflect the still-early phase of DAC technology and policy development and deployment. Given this, a key question in relation to DAC is whether we can anticipate public attitudes from attitudinal studies of CC(U)S, Bioenergy carbon capture and storage (BECCS) and geoengineering, which we consider below. In any case, as public knowledge and awareness of DAC is likely low,^13^ this will make attitudes labile, subject to influence by message and risk amplifiers such as NGOs and news organizations. A further strong influence can be expected to be prior attitudes relating to technology and environmental risk.^14^

There are many nationally specific studies of media framing of related technologies, e.g., for biochar, for which economic and innovation frames have been observed to predominate in UK news media,^15^ as in the present study. For CCS, national media attention cycles have been shown, for example, to have declined for Finland as of 2017^16^; to have been uncritically techno-optimist in Japan^17^; and in Germany, to be persistently associated with coal.^18^

Media frame analysis of geoengineering also initially found mostly generic frames.^19^ Conversely, public debate of BECCS in climate policy circles has found frames relating to values, scientific uncertainty and the integrity of science to be prevalent.^20^ Indeed Anselm and Hansson^21^ make the point that what particularly matters as discourses and storylines develop in relation to geoengineering, including those that are critical,^22^ are the political and other drivers of differing views. For DAC, this merits closer attention also, as do other details such as the persuasive or otherwise catalytic role of demonstration projects and their visibility.^23^

There is some research on the ways in which public support for geoengineering and climate intervention technologies, including DAC, differ across countries. Such research suggests a greater level of support for DAC in the Global South, and a higher level of support for ecosystem-based carbon dioxide removal in both North and South, relative to engineered removal.^24^ In the US, despite the possibility of moral hazard vis a vis carbon dioxide removal, in the sense of potentially reducing climate concern, survey evidence suggests rather that the US public is more concerned that polluters be held responsible for their carbon dioxide emissions, and that CDR projects should consult and engage people living close by.^25^ This emphasis on the procedural aspects of CDR deployment is also borne out in focus groups with US publics.^26^^,^^27^

The five generic news frames^8^ function here in their intended characterization role, with attribution of responsibility and economic consequences the most prevalent. However our analysis here does not extend to examining underpinning arguments and values. Nonetheless, of the frames that we observe, concerns about the lack of, and need for, governmental action on both climate change and DAC, and concerns about the financial viability of DAC, were notable. Moreover DAC is currently rarely framed in relation to conflict or morality, which helps to minimize polarization. Human interest frames have been little used to date but are potentially influential, for example if projects are undertaken with local community benefit. Positive impacts on employment, human health, and economy tend to be correlated with positive public perceptions,^28^ particularly if there are positive associations with human health.^29^

However, another implication of the early phase of DAC deployment and hence framing is that the differences between types of DAC technology are unlikely yet to be demarcated in the public mind.^30^ There is thus an argument for bottom-up or grounded inference of frames that are more specific to DAC and associated potential controversy. For example, this may relate to the perceived naturalness of the technology, which in addition to likely playing a role in perceptions of geoengineering^31^ and different CDR technologies,^32^^,^^33^^,^^34^^,^^35^ has also long been known as a feature of other technology acceptance.^36^ DAC methods that appear to be congruent with natural cycles may benefit from such association.^37^^,^^38^ As CCS has matured as a technology, ethical perspectives have gained prominence,^39^ and DAC has the same potential, with perhaps mixed consequences.

Overall, news media frame analysis can provide valuable insights into how DAC and other carbon capture technologies are perceived, and may be perceived in future. The approach thus provides a way of probing public perception of DAC technology. Currently, DAC appears to “get a better press” in North America than in Europe, but these are still early days. While conflictual, moral and human interest frames are relatively uncommon in relation to DAC, all have persuasive potential that both DAC advocates and skeptics might be advised to take note of. It would also be useful to conduct more controlled comparative analysis with specific newspapers, which should provide more reliability over time (please see the study limitations in the following text).

### Limitations of the study

It is important to note that Nexis databases are dynamic and subject to change over time, a function of Nexis’ choices and choices made by the managers of the databases of which Nexis is comprised. In practice this can and does complicate replicability. Replicability with the same outcome is more likely when sources are very tightly scoped (for example, by using specific newspapers), and when the period between repeat search is short. As an example of changes that compromise replicability, in September 2024, Nexis added 284 newspaper titles to its North American database (+43% relative to the original search). Hence pre- and post-09/24 searches give different (but overlapping) search returns. It may also be noted that two newspaper datasets dominate the North American results: while one is composed of geographically diverse sources, the other is composed of some 70 Canadian newspapers. This in turn leads to what might be considered a high proportion of Canadian articles in our sample (46%). These are not necessarily about Canadian DAC projects, but originate from (often syndicated) Canadian newspapers. In short, the ownership structure of the press, Nexis’ choices, and the choices made at the level of sub-databases do affect the search results with this type of work, something that we have not seen noted in media frame studies.

## Resource availability

### Lead contact

Further information and requests for resources should be directed to and will be fulfilled by the lead contact, Prof Paul J. Upham (p.j.upham@rug.nl).

### Materials availability

The newspaper sources were as available in Nexis Uni at the time of search. Tables of the newspaper articles returned at the time of search are appended. Repeat search at later dates will deliver a different article set due to database changes.

### Data and code availability

The news articles were systematically collected from a repository of news sources, Nexis Uni. The primary aim was to identify the news media frames relating to DAC in Europe and North America, initially from 1990 to 2023 and then scoped to the occurrence of the first news article in the sample, i.e., 2011. The Nexis Uni repository offers a wide range of news sources press releases, newspapers, newsletters, news transcripts, industry analyst reports, etc. on an international basis. Within the Nexis Uni repository, following criteria were applied when selecting articles.

• *Search term.* After a trial of search terms, the keywords were set simply as “direct air capture,” rather any more specific term. As DAC is still considered a novel and evolving technology,^3^ keeping the search term broad provided the most diverse and comprehensive set of news articles.
• *Time Period.* One of the secondary objectives was to trace the evolution of the way in which DAC has been framed over time. While the Nexis Uni repository dates back to 1980, the time period from 1990 and then 2011 to 2023 was selected, with articles on the topic only appearing in 2011 onwards.
• *Source type.* Of the various sources available in the database, only newspapers were selected. Newspapers are chosen as a form of text, as, relative to industry journals, for example, as they have a reputation for a degree of neutrality on news reporting^40^ and cover a wide range of topics and events. Nonetheless it is acknowledged that there are real differences in how and what different newspapers report, something that we did not probe here, except on a per continent basis.
• *Location.* Two datasets of news articles were created. Location by publication was selected for Europe in one set and North America in the other set.
• *Language.* The language filter was set to English for pragmatic reasons, with a single language preserving linguistic consistency. However this means that any differential reporting across languages cannot be observed.
• *Article word count.* No filter was applied regarding the maximum number of words per article.

Application of these filters in the Nexis Uni repository gave 659 news articles in Europe and 656 news articles in North America, totaling c.845,000 words. To create an attainable yet representative sample size, every 10th news article was sampled for further analysis, reducing the number of words to be manually read and coded to c.82,000. In case of the repetition of an article, or if an article was considered irrelevant, it was excluded manually. Articles were deemed irrelevant when DAC was only mentioned as a brief and incidental reference. The chronologically subsequent article was chosen as the substitute article in this case. Altogether, 64 articles were selected and analyzed for Europe, with a mean length of 843 words and median length of 585 words; 64 articles were selected for North America, with a mean length of 845 words and a median length of 700 words. All newspapers, regardless of their prominence, reputation or reach were considered, in order to create a diverse pool of news articles across different countries and journalists, albeit in English only.

Other items: Any additional information required to reanalyze the data reported in this paper is available from the lead contact upon request.

## Acknowledgments

We thank an anonymous reviewer for additional suggestions regarding contextual literature. No funding external to the University of Groningen was involved.

## Author contributions

E.I.: methodology, investigation, formal analysis, data curation, visualization, writing – original draft. P.J.U.: supervision, conceptualization, methodology, writing – original draft, review and editing.

## Declaration of interests

The authors declare no competing interests.

## STAR★Methods

### Key resources table

**Table:** 

REAGENT or RESOURCE	SOURCE	IDENTIFIER
**Software and algorithms**		

MS Office	Microsoft	
Nexis Uni	LexisNexis	

### Method details

A mixed-method approach was used to code and analyze the data. First, qualitative analysis consisted of manually assigning quotations from the news articles to pre-existing *a priori* frame codes relating to news media frame analysis. To briefly explain each frame code: *attribution of responsibility* portrays the cause or origin of an event or issue - focusing on the accountable party, whether this is an individual, group, or the government. *Human interest* highlights the personal or emotional aspect of an event, issue, or problem. *Conflict* emphasizes the disagreement between opposing parties to engage the interest of the audience. *Morality* evaluates events or issues in terms of ethical or societal standards. The *economic consequences* frame focuses on the economic impacts of an event, problem, or issue on an individual, group, institution, region, or country.

To make a finer distinction between these frames, various frame indicators [Table 1] were used to improve the coding process. Frame indicators are distinct elements that help to highlight the use of a particular frame.^41^ The frame indicators in Table 1 are based on selected frame indicators from Semetko and Valkenburg,^8^ adapted for the specific context of DAC. In Table 1, we use the word ‘concerns’ synonymously with ‘issues’, whether or not there is concern in the sense of anxiety, fear or perceived risk involved. This keeps the range of relevance open, within each prescribed frame indicator, hence avoiding loss of information that may be considered relevant in further work.

Using the frame indicators of Table 1, quotations from all sampled news articles were allocated manually. One article could contain multiple frame indicators and therefore overall frames. Allocation was on the basis of the face value interpretation and hence judgment of the coder. Quotations were also selected where particularly relevant, with relevance taking account of both context and thematic correspondence. Prior to the full analysis, a pilot analysis was performed with 20 randomly selected news articles. Here the coding scheme was tested, clarified and adjusted accordingly. After all articles were coded once, a second round of coding by the same coder was performed to maintain consistency, and to take into account the deeper understanding of the data gained after the full first round of coding. The Appendix provides a detailed list of sources for the quotations listed in the main paper, code quotations, and additional information

### Quantification and statistical analysis

Following the qualitative analysis, the presence or absence of frame indicators was manually assessed quantitatively for all articles sampled. Binary coding of the presence of a frame indicator was used: ‘1’ for presence, and ‘0’ for absence, enabling the production of line and bar charts for graphical representation. In addition, all articles were given codes for date of publication, country of publication, word count, newspaper source, speaker type, and tone of the article. The data for all Figures are appended.

As stated, application of filters gave 659 news articles in Europe and 656 news articles in North America, reduced as a 10% sample (with irrelevant articles removed) to 64 articles for Europe and 64 articles for North America. Descriptive statistics of simple numerical counts and percentages were calculated.

## Supplemental information

Document S1. Appendices A–C

Data S1. Results Europe

Data S2. Results North America

Data S3. Results Europe cf. North America

## References

[1] Schweizer V.J., Ebi K.L., van Vuuren D.P., Jacoby H.D., Riahi K., Strefler J., Takahashi K., van Ruijven B.J., Weyant J.P. (2020). Integrated Climate-Change Assessment Scenarios and Carbon Dioxide Removal. One Earth.

[2] Strefler J., Bauer N., Humpenöder F., Klein D., Popp A., Kriegler E. (2021). Carbon dioxide removal technologies are not born equal. Environ. Res. Lett..

[3] Sodiq A., Abdullatif Y., Aissa B., Ostovar A., Nassar N., El-Naas M., Amhamed A. (2023). A review on progress made in direct air capture of CO2. Environ. Technol. Innov..

[4] Sovacool B.K., Baum C.M., Low S. (2023). Reviewing the sociotechnical dynamics of carbon removal. Joule.

[5] IEA (2022).

[6] Goffman E. (1974).

[7] Neuman W.R., Just M.R., Crigler A.N. (1992).

[8] Semetko H.A., Valkenburg P.M.V. (2000). Framing European Politics: A Content Analysis of Press and Television News. J. Commun..

[9] IPCC (2022). Climate Change 2022: Mitigation of Climate Change. Contribution of Working Group III to the Sixth Assessment Report of the Intergovernmental Panel on Climate Change.

[10] Chen K., Molder A.L., Duan Z., Boulianne S., Eckart C., Mallari P., Yang D. (2022). How Climate Movement Actors and News Media Frame Climate Change and Strike: Evidence from Analyzing Twitter and News Media Discourse from 2018 to 2021. Int. J. Press.

[11] Cox E., Müller-Hansen F., Lamb W.F., Repke T., Bellamy R., S L., Smith S.M., Geden O., Gidden M.J., Lamb W.F., Nemet G.F., Minx J.C., Buck H., Burke J., Cox E., Edwards M.R. (2024). The State of Carbon Dioxide Removal - 2nd Edition.

[12] Repke T., Muller-Hansen F., Cox E., Minx J. (2023). Growing online attention and positive sentiments towards carbon dioxide removal.

[13] Cummings C., Lin S., Trump B.D. (2017). Public perceptions of climate geoengineering: a systematic review of the literature. Clim. Res..

[14] Brunsting S., De Best-Waldhober M., Terwel B.W. (2013). “I reject your reality and substitute my own!” Why more knowledge about CO2 storage hardly improves public attitudes. Energy Proc..

[15] Morris C., Price C., Nerlich B. (2024). Biochar in the UK Print News Media: Issue Frames and Their Implications for Opening up Debate About Land-based Greenhouse Gas Removal. Environ. Commun..

[16] Kojo M., Innola E. (2017). Carbon Capture and Storage in the Finnish Print Media. Risk, Hazards Cris. Publ. Pol..

[17] Asayama S., Ishii A. (2017). Selling stories of techno-optimism? The role of narratives on discursive construction of carbon capture and storage in the Japanese media. Energy Res. Social Sci..

[18] Koskinen O., Breyer C. (2018). Sustainability guardrails for energy scenarios of the global energy transition. Renewable and Sustainable Energy Reviews.

[19] Porter K.E., Hulme M. (2013). The emergence of the geoengineering debate in the UK print media: a frame analysis. Geogr. J..

[20] Haikola S., Hansson A., Anshelm J. (2019). From polarization to reluctant acceptance–bioenergy with carbon capture and storage (BECCS) and the post-normalization of the climate debate. J. Integr. Environ. Sci..

[21] Anshelm J., Hansson A. (2014). Battling Promethean dreams and Trojan horses: Revealing the critical discourses of geoengineering. Energy Res. Social Sci..

[22] Anshelm J., Hansson A. (2015). Has the grand idea of geoengineering as Plan B run out of steam?. Anthropol. Rev..

[23] Benner A.-K., Rothe D. (2024). World in the making: On the global visual politics of climate engineering. Rev. Int. Stud..

[24] Baum C.M., Fritz L., Low S., Sovacool B.K. (2024). Public perceptions and support of climate intervention technologies across the Global North and Global South. Nat. Commun..

[25] Scott-Buechler C. (2024). Removing carbon, restoring trust: public perceptions of industry and community roles in U.S. carbon dioxide removal policy.

[26] Scott-Buechler C., Cain B., Osman K., Ardoin N.M., Fraser C., Adcox G., Polk E., Jackson R.B. (2024). Communities conditionally support deployment of direct air capture for carbon dioxide removal in the United States. Commun. Earth Environ..

[27] Scott-Buechler C., Wang K., Fraser C., Scott C. (2024). Complex socio-technical transitions in fossil fuel country: considerations for direct air capture deployment in the.

[28] Steg L. (2023). Psychology of Climate Change. Annu. Rev. Psychol..

[29] Rocque R.J., Beaudoin C., Ndjaboue R., Cameron L., Poirier-Bergeron L., Poulin-Rheault R.-A., Fallon C., Tricco A.C., Witteman H.O. (2021). Health effects of climate change: an overview of systematic reviews. BMJ Open.

[30] Baum C.M., Fritz L., Low S., Sovacool B.K. (2024). Like diamonds in the sky? Public perceptions, governance, and information framing of solar geoengineering activities in Mexico, the United Kingdom, and the United States. Environ. Polit..

[31] Corner A., Parkhill K., Pidgeon N., Vaughan N.E. (2013). Messing with nature? Exploring public perceptions of geoengineering in the UK. Global Environ. Change.

[32] Bellamy R. (2022). Mapping public appraisals of carbon dioxide removal. Global Environ. Change.

[33] Cox E., Spence E., Pidgeon N. (2020). Public perceptions of carbon dioxide removal in the United States and the United Kingdom. Nat. Clim. Change.

[34] Wolske K.S., Raimi K.T., Campbell-Arvai V., Hart P.S. (2019). Public support for carbon dioxide removal strategies: The role of tampering with nature perceptions. Clim. Change.

[35] Müller-Hansen F., Repke T., Baum C.M., Brutschin E., Callaghan M.W., Debnath R., Lamb W.F., Low S., Lück S., Roberts C. (2023). Attention, sentiments and emotions towards emerging climate technologies on Twitter. Global Environ. Change.

[36] Siegrist M., Hartmann C. (2020). Consumer acceptance of novel food technologies. Nat. Food.

[37] Hilser H., Cox E., Moreau C., Hiraldo L., Draiby A., Winks L., Andrews M.G., Walworth N.G. (2024). Localized governance of carbon dioxide removal in small island developing states. Environ. Dev..

[38] Corner A., Pidgeon N. (2015). Like artificial trees? The effect of framing by natural analogy on public perceptions of geoengineering. Clim. Change.

[39] Gough C., Mander S. (2019). Beyond Social Acceptability: Applying Lessons from CCS Social Science to Support Deployment of BECCS. Curr. Sustain. Energy Reports.

[40] Franklin B. (2008). The Future of Newspapers. Journal. Pract..

[41] Linström M., Marais W. (2012). Qualitative news frame analysis: A methodology. Communitas.

