# Supplementary material for: Media frame development of direct air capture 2011–2023: A comparative analysis of Europe and North America
Source: iScience. 2024 Nov 12;27(12):111360. doi: 10.1016/j.isci.2024.111360 (PMC11616079; doi:10.1016/j.isci.2024.111360)
Supplement: Document S1. Appendices A–C [file mmc1.pdf]

iScience, Volume *27*

Supplemental information

Media frame development of direct air capture 2011–2023: A comparative analysis of Europe and North America

Paul J. Upham and Emina Ibrahimović

Appendices

Appendix A: European news article sample

(November 8, 2021 Monday). Air-scrubbing machines gain momentum, but long way to go. The Independent (United Kingdom). https://advance-lexis-com.proxyub.rug.nl/api/document?collection=news&id=urn:contentItem:641P-FSS1-JBNF-W06V-0000000&context=1516831.

Alan Young. (June 17, 2021 Thursday). Net zero talk perpetrates a global con – Richard Dixon. Scotsman. https://advance-lexis-com.proxyub.rug.nl/api/document?collection=news&id=urn:contentItem:62XX-2S91-JDPF-B119-0000000&context=1516831.

Andrew Woodcock. (November 17, 2020 Tuesday). Boris Johnson's green plan brings ban on petrol cars forward to 2030 and promises UK's first hydrogen-powered town. The Independent (United Kingdom). https://advance-lexis-com.proxyub.rug.nl/api/document?collection=news&id=urn:contentItem:619W-PPS1-DY4H-K28S-0000000&context=1516831.

Associated Press. (October 24, 2018 Wednesday). Giant fans that suck up greenhouse gases from the air must become commonplace across the US to fight climate change, report says. MailOnline. https://advance-lexis-com.proxyub.rug.nl/api/document?collection=news&id=urn:contentItem:5TJT-DHF1-F021-63SD-0000000&context=1516831.

Ben Spencer. (April 3, 2022 Sunday). 'Carbon extractor fans' only way to save the planet; Ben Spencer. The Sunday Times (London). https://advance-lexis-com.proxyub.rug.nl/api/document?collection=news&id=urn:contentItem:654R-WXM1-JCBW-N2SP-0000000&context=1516831.

Brad Plumer and Christopher Flavelle. (January 21, 2021 Thursday). Businesses Aim to Pull Greenhouse Gases From the Air. It's a Gamble.. The New York Times - International Edition. https://advance-lexis-com.proxyub.rug.nl/api/document?collection=news&id=urn:contentItem:61TF-8J71-DYR7-C17W-0000000&context=1516831.

(April 21, 2022 Thursday). Carbon removal: the 21st century's oil?. Global Capital Euroweek. https://advance-lexis-com.proxyub.rug.nl/api/document?collection=news&id=urn:contentItem:67BG-6DX1-JD35-D3SJ-0000000&context=1516831.

(September 9, 2023 Saturday). Collisons' carbon removal fund makes new commitments. Irish Examiner. https://advance-lexis-com.proxyub.rug.nl/api/document?collection=news&id=urn:contentItem:696F-7H21-DYRW-V42D-0000000&context=1516831.

Emma Gatten. (September 21, 2021 Tuesday). Carbon carbon everywhere, yet still a very tricky job to capture it; Analysis. The Daily Telegraph (London). https://advance-lexis-com.proxyub.rug.nl/api/document?collection=news&id=urn:contentItem:63NC-JW11-DYTY-C4ST-0000000&context=1516831.

Emma Newlands. (July 21, 2021 Wednesday). Decarbonisation-focused Scottish Cluster 'could create 20,000-plus jobs in next decade'. Scotsman. https://advance-lexis-com.proxyub.rug.nl/api/document?collection=news&id=urn:contentItem:6366-KGY1-JDPF-B3T1-0000000&context=1516831.

Hamish Penman. (August 31, 2021 Tuesday). Question mark over Direct Air Capture tech's viability. Aberdeen Press and Journal. https://advance-lexis-com.proxyub.rug.nl/api/document?collection=news&id=urn:contentItem:63GX-9Y41-JDMP-B0K8-0000000&context=1516831.

Ian Johnston. (August 21, 2017 Monday). World has missed chance to avoid dangerous global warming - unless we start geo-engineering the planet; Computer models predict average temperature will overshoot Paris Agreement targets but be brought back down again by the end of this century. The Independent (United Kingdom). https://advance-lexis-com.proxyub.rug.nl/api/document?collection=news&id=urn:contentItem:5P98-WHV1-JCJY-G533-0000000&context=1516831.

Jamie Hall. (July 9, 2020 Thursday). Appeal for Chancellor to support energy sector. Aberdeen Evening Express. https://advance-lexis-com.proxyub.rug.nl/api/document?collection=news&id=urn:contentItem:609V-5111-F13S-21MM-0000000&context=1516831.

John Collins Rudolf. (May 11, 2011 Wednesday). Carbon in the air: A target for ecologists; But study casts doubts on viability of capturing and sequestering it. The International Herald Tribune. https://advance-lexis-com.proxy-ub.rug.nl/api/document?collection=news&id=urn:contentItem:52V1-8361-JC85-N0TP-0000000&context=1516831.

John Reynolds. (August 25, 2019). Stripe to spend at least $1m a year on carbon capture tech; GREEN TECHNOLOGY. Sunday Independent. https://advance-lexis-com.proxyub.rug.nl/api/document?collection=news&id=urn:contentItem:5WWS-TPW1-JCBW-N0C600000-00&context=1516831.

Laura Roddy. (February 6, 2022 Sunday). Clean air start-up wins fresh funding. The Sunday Times (London). https://advance-lexis-com.proxyub.rug.nl/api/document?collection=news&id=urn:contentItem:64PT-PH71-DYTY-C4FX-0000000&context=1516831.

Leslie Hook. (February 2, 2019 Saturday). Could a superplant save the world?; Scientists are exploring whether an 'Ideal Plant' can help curb global warming. Leslie Hook meets them at their Californian lab ; Can a plant save the planet?. Financial Times (London, England). https://advance-lexis-com.proxyub.rug.nl/api/document?collection=news&id=urn:contentItem:5VB7-1FX1-JCBW-N0PV-0000000&context=1516831.

Luca Corradi. (July 26, 2021 Monday). Why net zero tech is the future - Luca Corradi. Scotsman. https://advance-lexis-com.proxyub.rug.nl/api/document?collection=news&id=urn:contentItem:636P-01H1-JDPF-B3X8-0000000&context=1516831.

Marc Gunther. (December 16, 2015 Wednesday). Startups have figured out how to remove carbon from the air. Will anyone pay them to do it?; Three startups, Carbon Engineering, Global Thermostat and Climeworks, are making strides with technology that can directly remove carbon dioxide from the air. What they need now is a viable business model. The Guardian. https://advance-lexis-com.proxyub.rug.nl/api/document?collection=news&id=urn:contentItem:5HMB-XSV1-F021-621P-0000000&context=1516831.

Myles McCormick. (November 1, 2021 Monday). Demand for 'climate tech' fuels investment boom; Capital raising More solutions, both incremental and disruptive, are needed to meet targets, writes Myles McCormick. Financial Times (London, England). https://advance-lexiscom.proxy-ub.rug.nl/api/document?collection=news&id=urn:contentItem:6404-HFV1-JCBWN2T4-00000-00&context=1516831.

(June 2021). Norwegian tomatoes go climate neutral. The Scottish Farmer. https://advancelexis-com.proxy-ub.rug.nl/api/document?collection=news&id=urn:contentItem:62WX-M0J1F0JC-M2DT-00000-00&context=1516831.

Oliver Wright. (July 3, 2020 Friday). Cummings wins £100m to save planet by sucking CO2 from air. The Times (London). https://advance-lexis-com.proxyub.rug.nl/api/document?collection=news&id=urn:contentItem:608G-R151-JCBW-N2XK-0000000&context=1516831.

Pilita Clark. (February 8, 2023 Wednesday). Windfall taxes not the only option for fossil fuel profits; Opinion Environment. Financial Times (London, England). https://advance-lexiscom.proxy-ub.rug.nl/api/document?collection=news&id=urn:contentItem:67H2-85N1-JCBWN3GR-00000-00&context=1516831.

(September 17, 2020 Thursday). Process will allow companies to make up for past emissions; DAC. The Press and Journal. https://advance-lexis-com.proxyub.rug.nl/api/document?collection=news&id=urn:contentItem:60VP-PB21-JCBW-N10T-0000000&context=1516831.

Rachel Millard. (March 3, 2021 Wednesday). Global investors stoke up on UK carbon capture project. The Daily Telegraph (London). https://advance-lexis-com.proxyub.rug.nl/api/document?collection=news&id=urn:contentItem:6249-WHF1-JCBW-N26P-0000000&context=1516831.

(April 26, 2021 Monday). Sucking it up Direct air capture of CO2. Financial Times (London, England). https://advance-lexis-com.proxyub.rug.nl/api/document?collection=news&id=urn:contentItem:62HT-4901-DYTY-C341-0000000&context=1516831.

(August 1, 2022 Monday). The secret of the philosopher's stone; Letters. Western Daily Press. https://advance-lexis-com.proxyub.rug.nl/api/document?collection=news&id=urn:contentItem:662B-RG61-JCBW-N2VV-0000000&context=1516831.

Appendix B: North American news article sample

(July 23, 2015 Thursday). A 'Third Way' to fight climate change. The Spec. https://advancelexis-com.proxy-ub.rug.nl/api/document?collection=news&id=urn:contentItem:5GHJ-SFG1JDV5-F1TN-00000-00&context=1516831.

(October 14, 2021). B.C. government puts $2M toward carbon capture fuel plant at Merritt. Abbotsford News. https://advance-lexis-com.proxyub.rug.nl/api/document?collection=news&id=urn:contentItem:63VC-YMK1-F0HF-84D3-0000000&context=1516831.

Bob Weber. (June 8, 2018 Friday). B.C. firm says it is sucking carbon from air, changing it to fuel. National Post's Financial Post & FP Investing (Canada). https://advance-lexis-com.proxyub.rug.nl/api/document?collection=news&id=urn:contentItem:5SH9-7WJ1-JBKR-R48W-0000000&context=1516831.

(January 28, 2020 Tuesday). Carbon Capture Gets Nudge In Joint Venture; Energy. National Post's Financial Post & FP Investing (Canada). https://advance-lexis-com.proxyub.rug.nl/api/document?collection=news&id=urn:contentItem:5Y31-K691-DY2T-F30S-0000000&context=1516831.

(April 7, 2022). Commentary: Will the world again hit 'snooze' on latest climate alarm?. Yukon News. https://advance-lexis-com.proxyub.rug.nl/api/document?collection=news&id=urn:contentItem:655P-W2D1-JD2C-J4WW-0000000&context=1516831.

David Campbell. (November 13, 2021 Saturday). Nuclear energy and net zero by 2050; COP26. The Telegraph-Journal (New Brunswick). https://advance-lexis-com.proxyub.rug.nl/api/document?collection=news&id=urn:contentItem:642P-GBS1-F084-C06P-0000000&context=1516831.

Derrick Penner. (September 11, 2021 Saturday). Research project proposes turning CO2 into stone under ocean floor. The Vancouver Sun (British Columbia). https://advance-lexiscom.proxy-ub.rug.nl/api/document?collection=news&id=urn:contentItem:63K8-78N1-DY2TD268-00000-00&context=1516831.

Dylan Short. (April 14, 2022 Thursday). Chrystia Freeland visits Calgary to promote carbon capture credit. Postmedia Breaking News. https://advance-lexis-com.proxyub.rug.nl/api/document?collection=news&id=urn:contentItem:6576-5YH1-JDK3-90XJ-0000000&context=1516831.

(July 14, 2023 Friday). Group of countries warns that carbon capture technologies cannot substitute for drastic emissions cuts. CE Noticias Financieras English. https://advance-lexiscom.proxy-ub.rug.nl/api/document?collection=news&id=urn:contentItem:68PK-D611-DYY902PS-00000-00&context=1516831.

Gwynne Dyer. (October 25, 2019 Friday). Aviation industry must innovate to stop 'flygskam'(flight shaming). Chatham Daily News. https://advance-lexis-com.proxyub.rug.nl/api/document?collection=news&id=urn:contentItem:5XBT-5JR1-DYKB-H50K-0000000&context=1516831.

(November 17, 2019 Sunday). Is The Edge Is Here; A rock-solid climate solution for CO2 International team works to advance technology that will turn a greenhouse gas into rock. Times Colonist (Victoria, British Columbia). https://advance-lexis-com.proxyub.rug.nl/api/document?collection=news&id=urn:contentItem:5XHP-HXG1-DY2T-113K-0000000&context=1516831.

Jason McBride Contributor. (July 13, 2022 Wednesday). How carbon market could help scale climate solutions; Accountants are now propelling corporations and governments toward meaningful environmental action. Waterloo Region Record (Ontario). https://advance-lexiscom.proxy-ub.rug.nl/api/document?collection=news&id=urn:contentItem:65XB-67H1-JDV5F264-00000-00&context=1516831.

Lisa Johnson. (January 15, 2022 Saturday). Province rolls out $30M to help boost carboncapture technology. Edmonton Journal (Alberta). https://advance-lexis-com.proxyub.rug.nl/api/document?collection=news&id=urn:contentItem:64J4-9T51-JBKR-12F9-0000000&context=1516831.

(October 4, 2021 Monday). Made-from-CO2 concrete, lululemons and diamonds spark investor excitement. Postmedia Breaking News. https://advance-lexis-com.proxyub.rug.nl/api/document?collection=news&id=urn:contentItem:63S6-50K1-JDK3-90M5-0000000&context=1516831.

Marisa Coulton and Meghan Potkins. (March 2, 2023 Thursday). Cleantech firms eye move to U.S.; Energy. National Post's Financial Post & FP Investing (Canada). https://advance-lexiscom.proxy-ub.rug.nl/api/document?collection=news&id=urn:contentItem:67NS-BC31-JBKRR4WT-00000-00&context=1516831.

Meghan Potkins. (April 7, 2022 Thursday). Trudeau proposes tax credit to cover 50% of carbon capture technology cost. Postmedia Breaking News. https://advance-lexis-com.proxyub.rug.nl/api/document?collection=news&id=urn:contentItem:655P-JBK1-F125-10JD-0000000&context=1516831.

Melissa Hank. (July 2, 2021 Friday). Putting squeeze on C02 emissions. Sarnia Observer. https://advance-lexis-com.proxyub.rug.nl/api/document?collection=news&id=urn:contentItem:6324-2S61-DYM7-40BK-0000000&context=1516831.

(December 15, 2021 Wednesday). Musk sets another goal: he wants SpaceX to turn CO2 from the atmosphere into fuel. CE Noticias Financieras English. https://advance-lexis-com.proxyub.rug.nl/api/document?collection=news&id=urn:contentItem:649R-WNW1-JBJN-M21S-0000000&context=1516831.

Navdeep Bains. (September 17, 2022 Saturday). Keeping up with the Bidens; Canada needs to up its climate change game. National Post's Financial Post & FP Investing (Canada). https://advance-lexis-com.proxyub.rug.nl/api/document?collection=news&id=urn:contentItem:66DC-1CN1-DY2T-F2W9-0000000&context=1516831.

(May 19, 2021 Wednesday). New technologies alone will not solve climate crisis. CE Noticias Financieras English. https://advance-lexis-

com.proxyub.rug.nl/api/document?collection=news&id=urn:contentItem:62PY-VSW1-DY1R-B4FT-0000000&context=1516831.

(April 8, 2022). Ottawa unveils $2.6B carbon capture tax credit in budget. The Canadian Press. Red Deer Advocate. https://advance-lexis-com.proxyub.rug.nl/api/document?collection=news&id=urn:contentItem:655V-GBG1-JD2C-J3G6-0000000&context=1516831

Rebecca Gao. (June 10, 2023 Saturday). Is Canada too cold to capture carbon?; Ottawa-based company is working on new system that doesn't use liquids that easily freeze. The Toronto Star. https://advance-lexis-com.proxyub.rug.nl/api/document?collection=news&id=urn:contentItem:68F4-1451-JDV5-F4R1-0000000&context=1516831.

Rebecca Gao. (September 11, 2023 Monday). Tech Update: Canadian carbon-capture solutions are flying high. thestar.com. https://advance-lexis-com.proxyub.rug.nl/api/document?collection=news&id=urn:contentItem:6951-M1S1-F197-5057-0000000&context=1516831.

Robert Williams. (November 23, 2022 Wednesday). University of Waterloo receives $16 million to fund six projects on climate change. The Record (Waterloo Region, Ontario) Online. https://advance-lexis-com.proxyub.rug.nl/api/document?collection=news&id=urn:contentItem:66XV-9PH1-JDV5-F0KW-0000000&context=1516831.

(February 4, 2021 Thursday). Scared by global warming? In Iceland, one solution is petrifying. National Post (f/k/a The Financial Post) (Canada). https://advance-lexis-com.proxyub.rug.nl/api/document?collection=news&id=urn:contentItem:61XM-H5G1-JBKR-G0KG-0000000&context=1516831.

(December 13, 2020 Sunday). Slowing Climate Change With Sewage Treatment for the Skies. Postmedia Breaking News. https://advance-lexis-com.proxyub.rug.nl/api/document?collection=news&id=urn:contentItem:61H9-7G01-F125-12VT-0000000&context=1516831.

(January 11, 2019 Friday). Smith: Let's celebrate that Canada is likely a 'net zero' polluter. Postmedia Breaking News. https://advance-lexis-com.proxyub.rug.nl/api/document?collection=news&id=urn:contentItem:5V5K-HW11-JDK3-9345-0000000&context=1516831.

(October 17, 2023 Tuesday). Technological innovation, the key to climate change?. CE Noticias Financieras English. https://advance-lexis-com.proxyub.rug.nl/api/document?collection=news&id=urn:contentItem:69DV-68W1-JCG7-81CT-0000000&context=1516831.

(February 20, 2021 Saturday). The challenge of zeroing emissions. CE Noticias Financieras English. https://advance-lexis-com.proxyub.rug.nl/api/document?collection=news&id=urn:contentItem:6227-2J91-DY1R-B381-0000000&context=1516831.

Tiffany Crawford. (August 12, 2023 Saturday). B.C. Climate News: Deadly wildfire destroys town in Maui. Postmedia Breaking News. https://advance-lexis-com.proxyub.rug.nl/api/document?collection=news&id=urn:contentItem:68XK-J7C1-JCDT-J43T-0000000&context=1516831.

(December 10, 2020 Thursday). United Will Suck Carbon From the Air Instead of Buying Offsets. National Post (f/k/a The Financial Post) (Canada). https://advance-lexis-com.proxyub.rug.nl/api/document?collection=news&id=urn:contentItem:61GP-MN81-JBKR-G1N4-0000000&context=1516831.

(August 12, 2021 Thursday). With New Urgency, Climate Scientists Recommend Carbon Removal. National Post (f/k/a The Financial Post) (Canada). https://advance-lexis-com.proxyub.rug.nl/api/document?collection=news&id=urn:contentItem:63BX-5XV1-JBKR-G1BW-0000000&context=1516831.

(September 8, 2021 Wednesday). World's largest carbon-capture plant by Climeworks starts making tiny dent in emissions. National Post (f/k/a The Financial Post) (Canada). https://advance-lexis-com.proxyub.rug.nl/api/document?collection=news&id=urn:contentItem:63JP-HXD1-DY2T-60GH-0000000&context=1516831.

Yadullah Hussain. (January 21, 2022 Friday). Canada needs policy clarity around carbon; Energy. National Post's Financial Post & FP Investing (Canada). https://advance-lexiscom.proxy-ub.rug.nl/api/document?collection=news&id=urn:contentItem:64KF-4C71-DY2TF2KC-00000-00&context=1516831.

Appendix C Exemplar quotations by media frame type and indicator

Illustrative quotations: attribution of responsibility, Europe

Does the story suggest that some level of the government has the capacity to address DAC-related concerns?

1	“Carbon Engineering, Global Thermostat and Climeworks all sprung up during the mid-to-late-2000s, when it looked as if the world's governments might take aggressive action to curb climate change. Mostly, they haven't.” (The Guardian, 2021)
2	“Regular readers will be familiar with criticism of governments, including our own, which tell us that carbon capture and storage and related technical fixes will be the answer, when they are a decade away at any scale, if they happen at all.” (The Scottish Farmer, 2021)
Does the story suggest that an individual (or group of people in society) is responsible for DAC-related concerns?	
3	“But as global warming accelerates and society continues to emit greenhouse gasses at a dangerous rate, the idea is gaining support from a surprising source: large companies facing pressure to act on climate.” (The New York Times - International Edition, 2021)
4	“Direct air capture (DAC) technology has been likened to "time travel" as it lets companies take responsibility for and scrub out past emissions.” (The Press and Journal, 2020)
Does the story suggest strategies or actions to address DAC-related concerns?	
5	"DAC will provide a mechanism for those businesses to reduce their climate impact effectively and allow others to remove from the atmosphere the emissions that they were responsible for in the past." (The Press and Journal, 2020)
6	“Storegga's subsidiary Pale Blue Dot is developing the Acorn project, which will stash carbon dioxide emissions under the North Sea off the Scottish coast and produce hydrogen from natural gas. The new funds will help get Acorn to final investment decision, as well as help Storegga's plans for direct air capture (scrubbing CO2 from the air) and other low carbon projects.” (The Daily Telegraph, 2021)
Does the story suggest that DAC-related concerns require urgent action?	
7	‘"It's existential for us," said Peter Reinhardt, co-founder of Charm Industrial, a start-up that Stripe is paying to remove 416 tons of carbon dioxide at $600 per ton. His company will take crop waste and convert it into an oil that can be injected underground, rather than letting the waste decay and release carbon back into the atmosphere.’ (The New York Times - International Edition, 2021)
8	“Scientists previously thought limiting global warming to 2C would avoid the most dangerous effects, but there is increasing evidence that allowing it to go much above 1.5C could lock in considerable sea level rise for the next few centuries.” (The Independent, 2017)

Illustrative quotations: attribution of responsibility, North America

Does the story suggest that some level of the government has the capacity to address DAC-related concerns?	
9	“And governments must do more to support structural change through regulatory frameworks and financial incentives.” (CE Noticias Financieras English, 2021)
10	“Capturing CO2 is a less enticing prospect for many investors, who think the government should fund such expensive, high risk projects.” (Postmedia Breaking News, 2021)
Does the story suggest that an individual (or group of people in society) is responsible for DAC-related concerns?	
11	‘"We're going to still be emitting carbon," he said. - Chief Executive Officer Scott Kirby"’ (National Post (f/k/a The Financial Post, 2020)
12	“But technology will not solve the climate crisis for us. We also need to create an appropriate policy environment. A key component in the green transition will be the highest carbon pricing, which requires coordination and support at the international level.” (CE Noticias Financieras English, 2021)
Does the story suggest strategies or actions to address DAC-related concerns?	
13	“Sean Wilson, the CEO of Canadian DAC startup TerraFixing, says that the Intergovernmental Panel on Climate Change (IPCC) has identified DAC as one way the world can mitigate climate change. "They say we need about five gigatons of any carbon dioxide removal method, like direct air capture, by 2050," he says, which might help shed some light on DAC's recent popularity. Wilson also notes that the Canadian government's carbon capture investment tax credit has given the industry a boost.” (The Star, 2023)
14	‘"These policies will all be helped by expanding the knowledge base and capacity around CCUS opportunities, and by a roster of potential projects that can rapidly proceed once they come into effect," said Environment and Parks Minister Jason Nixon’ (Edmonton Journal, 2022)
Does the story suggest that DAC-related concerns require urgent action?	
15	“It's an inevitable fact that we'll be adding more CO2 to the atmosphere before we can achieve a carbonneutral technological base. Given the severe danger, we need to try every possible mitigation.” (Postmedia Breaking News, 2020)
16	‘“This is critically important," Kate Moran, project lead for the initiative under the name Solid Carbon, said about carbon-capture technology. "By the middle part of the century or earlier, it has been demonstrated by the science community that we need to be removing CO2 from the atmosphere in order to keep the planet habitable."’(The Vancouver Sun, 2021)

Illustrative quotations: human Interest, Europe

Does the story emphasize how individuals and groups are affected by DAC-related concerns?	
18	“It's not just about creating jobs, it's about protecting the thousands of jobs we know will be at risk.” (Aberdeen Evening Express, 2020)
19	“It has been calculated that the cluster, by deploying CCS, hydrogen and direct air capture (DAC) technologies in Scotland, could support an average of 15,100 workers between 2022 and 2050 – both
	directly and in the supply chain – while total such jobs are expected to peak at 20,600 in 2031.” (Scotsman, 2021)
Does the story acknowledge the diversity of opinions and perspectives within the DAC-related concerns?	
20	“Storegga, which is behind the Acorn carbon capture and storage project, and Carbon Engineering (CE) is forging ahead with plans for a north-east facility, which could remove up to one million tonnes of carbon dioxide from the atmosphere a year. But DAC has been served a reality check by Westwood Global Energy Group, which claims the technology might only make financial sense for firms in a handful of countries.” (Aberdeen Press and Journal, 2021)

Illustrative quotations: human Interest, North America

Does the story emphasize how individuals and groups are affected by DAC-related concerns?	
21	“Procrastination is no longer an option. The longer we delay, the more disruption we’ll cause, and the costlier it will become. Shifting away from coal, oil and gas and protecting nature will generate numerous benefits, from cleaner air and water to better jobs, health and economies.” (Yukon News, 2022)
22	‘"It's critical for us to be working toward net-zero," said Charmaine Dean, vice-president of research and international at Waterloo. "Using established and emerging strengths in fundamental and applied research, Waterloo is pushing boundaries to accelerate the transition to a climate-resilient, low-carbon sustainable society, and advancing the sustainable use and management of space, land, water and energy on a global scale."’(The Record, 2022)
23	“While Canada has implemented an investment tax credit aimed at alleviating some of the capital cost burden in building carbon capture projects, the measure doesn't shield investors from potential changes in future carbon prices. The U.S., on the other hand, has rules in place that guaranteed a price for carbon offsets generated by eligible projects over more than a decade, effectively de-risking large investments in carbon capture and storage.” (National Post (f/k/a The Financial Post, 2020)

24	“With Gates' support, Direct Air Capture, a company dedicated to the development of technology for absorbing carbon dioxide from the atmosphere, has secured contracts worth $3.5 billion dollars in the United States. However, this technology has generated criticism from environmentalists, who argue that the resources could be allocated to other solutions that would truly transform the energy and production system. Instead, they are a distraction on the road to a fossil fuel-free era. On the other hand, some believe that these technologies could play a crucial role during the transition to low-carbon economies until alternative energy and fuels become dominant.” (CE Noticias Financieras English, 2023)

Illustrative quotations: conflict, Europe

Does the story reflect disagreement between parties- individuals-groups-countries?	
25	“Climate activists have often been critical of companies and politicians placing blind faith in "silver bullet" solutions at the expense of the buildout of available technology such as wind and solar. In May, John Kerry, the climate envoy of US president Joe Biden, came under fire for saying half of the reductions necessary to hit net zero "are going to come from technologies we don't yet have".” (Financial Times, 2021)
26	“Some reputable scientists are highly sceptical of the concept for this reason. Current extraction technology is capable of taking out only a speck of carbon being emitted. This year, emissions in excess of 33 billion tonnes of carbon are expected from human activity, while the most advanced direct air capture (DAC)
	technology can remove only 4,000 tonnes of carbon annually.” (Western Daily Press, 2022)
27	“This has bolstered interest in direct air capture companies that suck carbon dioxide out of clean air rather than clouds of factory pollution. But none of these developments guarantee that global emissions will fall as quickly as they must. "And also, why should cash-strapped taxpayers pay for this when the industry is making out like bandits?" says Oxford university's Professor Myles Allen, who has spearheaded the carbon takeback idea.” (Financial Times, 2023)
Does the story refer to two sides or to more than two sides of the DAC-related concern?	
28	“Using technology to suck carbon dioxide out of the sky has long been dismissed as an impractical way to fight climate change - physically possible, but far too expensive to be of much use. But as global warming accelerates and society continues to emit greenhouse gases at a dangerous rate, the idea is gaining support from a surprising source: large companies facing pressure to act on climate.” (The New York Times - International Edition, 2021)
29	“Leading scientific agencies including the United Nations Intergovernmental Panel on Climate Change say that even if the world manages to stop producing harmful emissions, that still won't be enough to avert a climate catastrophe. They say we need to suck massive amounts of carbon dioxide out of the air and put it back underground - yielding what some call "negative emissions”.” (The Independent, 2021)

30	'"The main obstacle to the growth of CCUS, Budinis said, is that it is very energy-intensive - and hence costly. "That doesn't mean it doesn't work," she said. For DAC, she identified a second barrier: "a nonexistent supply chain for the components which are needed." - Sara Budinis, Energy Analyst at the International Energy Agency in Paris" (Global Capital Euroweek, 2022)
31	'"Recognising and supporting the potential of new and innovative solutions in their early stages of development, and the climate entrepreneurs behind them, is critical because no solution will singlehandedly reverse climate change," said Stacy Kauk, head of sustainability at Shopify, in a statement.' (Irish Examiner, 2023)

Illustrative quotations: conflict, North America

Does the story reflect disagreement between parties- individuals-groups-countries?	
32	"It's not possible to get actual carbon emissions to zero. And so the only way you can stop and reverse this is through direct air capture and sequestration. And that has seemed obvious to me for a long time. I think it is obvious to anyone that takes the time to understand the science and understand the math." (National Post (f/k/a The Financial Post, 2020)
33	"If environmentalists were genuinely worried about impending doom, they'd start having their meetings on FaceTime." (Postmedia Breaking News, 2019)
Does the story refer to two sides or to more than two sides of the DAC-related concern?	
34	"It is critical that the main focus of the Paris meeting remains on reducing the burning of fossil fuels' vs As the years go by, it will become highly desirable to remove some of that CO2." (The Spec, 2015)
35	"Direct air capture', a technology that has not been sufficiently tested and still has a high cost, is however, one of the developments in which the author states 'they will be fundamental' to eliminate gases that have already been emitted. - Bill Gates" (CE Noticias Financieras English, 2021)
36	"To keep or improve on this trajectory would likely mean major investments in technology. But not everyone agrees that's where our money should go." (National Post (f/k/a The Financial Post, 2021)
37	"Still, activists argue that focusing too much on carbon-removal technologies could become a distraction from the work of immediately reducing emissions." (National Post (f/k/a The Financial Post, 2021)
38	"When the budget was announced last week, several critics stated the government was contradicting itself by setting ambitious emission reduction goals while also scaling up the oil and gas industry. Freeland said Thursday that her government believes they can "walk and chew gum at the same time" and will be able to reduce emissions while continuing to grow the economy." (Postmedia Breaking News, 2022)

Illustrative quotations: morality, Europe

Does the story offer specific societal values or ethical guidelines when considering DAC-related decisions?	
40	“Everybody claims to be green these days but what the planet needs is actual zero carbon emissions, not the messy nightmare that is net zero.” (Scotsman, 2021)
41	‘"Now, you have the fundamental underlying economics, you have wonderful products, and you have a public acceptance that will increasingly push towards the net zero global economy," says Hans Kobler, managing partner of Energy Impact Partners, an investment firm with $2bn under management.’ (Financial Times, 2021)
42	‘"We have already failed on climate to the extent to which direct air capture is one of the many things we must do," Friedmann said. "We have already emitted so many greenhouse gases at such an incredible volume and rate that CO2 removal at enormous scales is required, as well as reduction of emissions.”’ (The Independent, 2021)
43	‘“Professor Shaun Fitzgerald, director of the Centre for Climate Repair at Cambridge University, argued that a reduction in emissions alone cannot be sufficient to stay within the 1.5C limit. He insists we must also remove greenhouse gases: "There's no point in emptying the bath if you keep topping it up."’(The Sunday Times, 2022)

Illustrative quotations: morality, North America

Does the story offer specific societal values or ethical guidelines when considering DAC-related decisions?	
45	“It is critical that the main focus of the Paris meeting remains on reducing the burning of fossil fuels. There is therefore a moral hazard in urging action on the third way. But knowing how long it takes to get new technologies to scale, and how soon we are likely to need them, there is also a moral hazard in ignoring the urgent need for immediate investment.” (The Spec, 2015)
46	“If we are all to feel like we belong in Canada, we need a common strategy that doesn't pit one province against the other, one industry against the other, and one Canadian against the other.” (Postmedia Breaking News, 2019)
47	“Another important aspect is ensuring that the venture has societal acceptance. A team from UBC will look at the social and regulatory needs of the project, including both a small-scale demonstration and a larger commercial-scale operation.” (Time Colonist, 2019)
48	“The solution here is simply to consider this technology a public utility creating a public good, like roads, national defense, fresh water, or sewage disposal-and pay for it as such.” (Postmedia Breaking News, 2020)

Does the story discuss consequences of certain actions or choices in terms of right and wrong?	
49	"A big DAC initiative, one that matched a significant percentage of the need, might require as much as a few percentage points of all the electricity generated. This sounds extreme, but consider that right now 2% of the world electricity supply is wasted on the creation of Bitcoin. It's possible that a similar amount directed to saving civilization could be considered reasonable." (Postmedia Breaking News, 2020)
50	"This incidentally offers a good benchmark for a global carbon price: If it costs $100 to remediate a ton of carbon emitted, then surely the polluter should pay an equivalent amount to emit it." (The Spec, 2015)

Illustrative quotations: economic consequences, Europe

Is there a mention of financial losses or gains now or in the future?	
51	"Several billionaires have adopted CO2 removal as a sort of pet project but it hasn't yet received significant government funding. Richard Branson launched a $25m global contest for CO2 removal technology in 2007. But even after more than a decade, no solution has been found." (Financial Times, 2019)
52	"If there was scalable, verifiable negative emissions technology available in the vicinity of $100 per tonne of CO2 captured, it could be a trillion-dollar industry by the end of the century, and complement emissions reduction in halting anthropogenic climate change." (Sunday Independent, 2019)
53	"Carbon Collect, a start-up that removes carbon dioxide from the air and has the former taoiseach Enda Kenny as a director, has closed a funding round worth more than (EURO)10 million." (The Sunday Times, 2022)
Is there a mention of the costs/degree of expense involved?	
54	"The report concluded that it would cost at least $600 a ton to capture carbon dioxide from the air, compared with an estimated cost of about $80 a ton to capture the gas from a typical coal power plant." (The International Herald Tribune, 2011)
55	"Carbon Engineering, a Canadian-based clean energy company backed by Bill Gates, recently outlined the design of a large industrial plant that it said could capture carbon dioxide from the atmosphere at a cost of between $94 and $232 a tonne.That is well below past estimates of about $600 a tonne by the American Physical Society, said David Keith, a Harvard University physics professor and the founder of Carbon Engineering who led the research." (Associated Press, 2018)
56	"At least two companies have now created working direct air capture plants but at the moment it costs nearly £500 to remove a single tonne of CO2." (The Times, 2020)

57	“The hope, companies say, is that early investments can help drive down prices to something more palatable - say, $100 per ton or less - much as investments in wind and solar have made those energy sources cheaper over time.” (The New York Times - International Edition, 2021)
58	“Estimates vary, but it currently costs about $500 to $600 per ton to remove carbon dioxide using direct air capture, said Colin McCormick, chief innovation officer at Carbon Direct, which invests in carbon removal projects and advises businesses on buying such services.” (The Independent, 2021)
Is there a reference to economic consequences of pursuing or not pursuing a course of action?	
59	“What's more, if direct air capture of CO2 is to emerge as a meaningful climate solution, it would have to be built out at a global, industrial scale, costing billions of dollars.” (The Guardian, 2015)
Does the story consider economic viability/feasibility regarding the implementation of DAC?	
60	“Other analysts had mixed views. In an e-mail, Sasha Mackler, director for energy innovation at the Bipartisan Policy Center, a Washington institute, agreed that direct air capture of carbon dioxide was probably decades away from making economic sense.” (The International Herald Tribune, 2011)
61	“Emerging carbon capture methods like direct air capture, and enhanced capture in plants or through mineralisation, are "an opportunity for Stripe and like-minded early adopters to shift the trajectory of the industry, who ... can help by buying in early. If a broad coalition of buyers commits substantial investment, we're optimistic that the price curve will start to move", he added.” (Sunday Independent, 2019)
62	‘“But he warned: “Gaps still remain. Onshore wind and solar energy remain unsupported, long shots such as modular nuclear power and direct air capture may not pay off, and natural solutions to climate change – planting trees and restoring peat bogs – remain largely overlooked and ignored.”’ (The Independent, 2020)
63	“The International Energy Agency says the technology has "significant potential to accelerate the transition to net zero, but costs need to come down" from the current $135-$345 per tonne of CO2 captured and stored. Simpler methods, such as tree planting, can cost as little as $10 per tonne.” (Financial Times, 2021)
64	“What we need now is for the cost to significantly come down and the scale to go up; investing in technology innovation is key to achieve that. The UK has the opportunity to create a leading net zero supply chain, to decarbonise domestic industrial production and export technology to other countries.” (Scotsman, 2021)
65	“Major economies, including the UK, are pouring billions into research and development, but estimated costs vary widely and it may be some time before either carbon removal or carbon capture are cost competitive.” (The Daily Telegraph, 2021)

Illustrative quotations: economic consequences, North America

Is there a mention of financial losses or gains now or in the future?	
66	"For now the political pendulum in Washington seems to have shifted towards favoring carbon removal. In the bipartisan infrastructure bill the Senate passed this week, there was a record $9 billion plus for carbon capture. Deich notes the bill has $3.5 billion for four regional direct air capture hubs and another $3.5 billion for transport and storage of carbon including money to build a massive pipeline network to carry captured CO2." (National Post (f/k/a The Financial Post, 2021)
67	"Energy Minister Bruce Ralston and Upper Nicola Band Chief Harvey McLeod announced the project Thursday, with $2 million from the province's clean energy fund for engineering and design. Carbon Engineering, which operates a CO2 capture pilot project at Squamish, is a partner in the venture with Huron Clean Energy, a new company established in 2019 to develop "air to fuels" plants." (Abbotsford News, 2021)
68	"Musk, the world's richest man thanks to the popularity of his electric vehicle maker Tesla, said in January that he would donate $100 million to a prize for the best carbon capture technology. The new initiative to make fuel for spacecraft would be based on a type of technology, direct air capture (DAC), which is still in the early stages of development." (CE Noticias Financieras English, 2021)
69	"Alberta will spend $30 million to help speed up carbon capture design and engineering work, the UCP government said Friday." (Edmonton Journal, 2022)
70	"The Alberta government and the energy industry are seeking investment tax credits (ITC) of as much as 45 per cent from Ottawa to help fund the high cost of developing CCUS." (National Post (f/k/a The Financial Post, 2022)
71	"The Trudeau government's new fiscal plan also includes a 60-per-cent credit for investment in direct air capture technologies to remove carbon from the atmosphere. Direct air capture projects are far less common than CCUS, which is focused on retrofitting fossil fuel-based power and industrial plants to capture emissions at their source." (Postmedia Breaking News, 2022)
72	"New American credits, loans and incentives should hasten the deployment of existing climate technologies such as wind and solar and encourage more advanced technologies. For direct air capture (DAC), instead of the tax credit of $50 per ton of captured carbon, the government now offers $180 with an easier qualification threshold." (National Post (f/k/a The Financial Post, 2022)
73	"This work has garnered the attention of Klarna, a fintech company that's contributing a total of $2.35 million (U.S.) toward new and promising climate solutions around the world. TerraFixing is planning to use the funding to accelerate its tech development and compete in the high-profile $100-million Xprize for carbon removal, where it is one of the top 60 finalists." (The Toronto Star, 2023)
74	"Last April, Joe Biden's government announced grants of US$ 3.5 billion to build factories that will capture and permanently store carbon through Direct Air Capture (DAC)." (CE Noticias Financieras English, 2023)

75	“The Energy Department announced Friday it is awarding up to $1.2 billion to two projects to directly remove carbon dioxide from the air in what officials are calling the largest investment in "engineered carbon removal" in history.” (Postmedia Breaking News, 2023)
Is there a mention of the costs/degree of expense involved?	
76	“Carbon Engineering's fuel costs about 25 per cent more than gasoline made from oil. Oldham said work is being done to reduce that.” (National Post (f/k/a The Financial Post, 2018)
77	“The goal for Climeworks: Cut costs for capturing CO2 from ambient air to about US$100 a ton from around US$600 now. Combining the companies' systems will "create a game changer in the industry to be able to do direct-air capture at a smaller-sized plant," Svante CEO Claude Letourneau said.” (National Post (f/k/a The Financial Post, 2020)
78	“U.S. payments company Stripe said last year it would pay Climeworks $775 a tonne for extracting 322 tonnes of carbon dioxide from the air - one indication of the cost.” (National Post (f/k/a The Financial Post, 2021)
79	“University researchers want to test the idea that large amounts of carbon dioxide could be captured from the air offshore and injected into basalt aquifers deep beneath the ocean floor where it will solidify, essentially into stone. A demonstration project won't be cheap, $30 million to $60 million, but the consortium, which includes the University of Victoria, wants to figure out if this could be a game-changing technology in the race to stall climate change at 1.5 C of warming.” (The Vancouver Sun, 2021)
80	“The industry has said the large-scale buildout of carbon capture and storage in Canada will be contingent on government help. Energy producers had lobbied for a carbon capture tax credit to cover up to 75 per cent of the capital costs of investing in the expensive technology.” (The Canadian Press, 2022)
Is there a reference to economic consequences of pursuing or not pursuing a course of action?	
81	“But just as costs have come down as investments have been made in wind and solar, so the costs of thirdway technologies can be expected to fall over time.” (The Spec, 2015)
82	“United, despite unprecedented losses during 2020, has the resources to address emissions more directly than by purchasing offsets. "Part of the reason others haven't done it is because it does cost money to do this," he said. "If instead you can write a check to an NGO, and say you have offset all your carbon by writing a check, that's been historically an easier way to get there."” (National Post (f/k/a The Financial Post,
	2020)
Does the story consider economic viability/feasibility regarding the implementation of DAC?	
83	“It costs Climeworks about US$600 a tonne to remove carbon from the atmosphere. Carbon Engineering says it can do the job for between US$94 and US$232 a tonne because it uses technology and components that are well understood and commercially available.” (National Post (f/k/a The Financial Post, 2018)

84	“Scaling production up would take a long time and cost a lot, but it would also bring the price down to a commercially viable level.” (Chatham Daily News, 2019)
85	“Two companies that come at carbon capture differently will be working together with a goal to lower the cost of the technology by about 80 per cent, making it more widely accessible in the fight against climate change.” (National Post (f/k/a The Financial Post, 2020)
86	“Investing in direct-air capture, the logic goes, allows United to become an early champion of a technology that may become less expensive and more common as years tick by.” (National Post (f/k/a The Financial Post, 2020)
87	“These new technologies and processes can start at a high price. But as we've seen with solar panels and fuel cells, the costs of a technology tend to drop as soon as its use begins to increase.” (CE Noticias Financieras English, 2021)
88	“Since it needs emissions produced by other industries, it's not actually removing carbon dioxide from the air - it's just helping reduce additional CO2 from being emitted. That's where direct air capture (DAC) companies, which can capture CO2 from the sky, come in. Once their products are more affordable and more widely used, concrete could aid in actively lowering global CO2 emissions.” (Sarnia Observer, 2021)
89	“The catch with carbon-capture technologies is cost, said Chris Severson Baker, Alberta director for the energy think-tank the Pembina Institute. "The problem with these types of things is not that it isn't technologically feasible, (it's that) it's so expensive to do," Severson Baker said. Carbon capture is sometimes viewed as a distraction that gets in the way of cutting greenhouse-gas emissions, but Severson Baker said scientists acknowledge emissions reductions alone won't get countries to the goal of net zero by 2050.” (The Vancouver Sun, 2021)
90	“Further, the IEA concludes that to reach net zero emissions by 2050, nearly half (45 per cent) of the reduction will be based on technologies that are still under development. In other words, the IEA can't even say for sure how we are going to get there. The agency is calling for $90 billion (USD) to be spent immediately to accelerate research in areas such as advanced batteries, hydrogen electrolysers and direct air capture and storage.” (The Telegraph Journal, 2021)
91	‘"You need to stimulate ambition," says Lisa DeMarco, the chief executive of Resilient LLP and the International Emissions Trading Association council chair. "Our problem hasn't been that the solutions don't exist. The problem has been scoping and scaling them. With environmental markets and trading, you can get a trillion more dollars of action per year."’ (Waterloo Region Record, 2022)
92	“Under current conditions, Entropy's Belenkie said most carbon capture projects in Canada will take approximately 10 years to pay for themselves - and that payout isn't assured thanks to the political risk that governments could alter carbon prices or enact policies to make sequestration more costly.” (National Post (f/k/a The Financial Post, 2023)
